# Sodium Butyrate Ameliorates Gut Microbiota Dysbiosis in Lupus-Like Mice

**DOI:** 10.3389/fnut.2020.604283

**Published:** 2020-11-11

**Authors:** Hanchang He, Haoming Xu, Jing Xu, Hailan Zhao, Qianyun Lin, Youlian Zhou, Yuqiang Nie

**Affiliations:** ^1^Department of Nephrology, The First People's Hospital of Foshan, Foshan, China; ^2^Department of Medicine, Beth Israel Deaconess Medical Center, Harvard Medical School, Boston, MA, United States; ^3^Department of Gastroenterology, Guangzhou Digestive Disease Center, Guangzhou First People's Hospital, School of Medicine, South China University of Technology, Guangzhou, China

**Keywords:** gut microbiota, butyrate, systemic lupus erythematosus, lupus, MRL/lpr

## Abstract

Gut microbiota has a strong influence on the onset and development of systemic lupus erythematosus (SLE), and several studies have demonstrated the effectiveness of microbiota-derived butyrate to ameliorate SLE. However, the roles of butyrate on gut microbiota in SLE are not understood. Using MRL/lpr lupus-prone mice, we examined gut microbiota profiles after butyrate treatment by 16S rRNA sequencing. Alterations in intestinal microbiome in mice with lupus-like disease were mainly characterized by a reduction in microbial diversity, with an increased abundance of Bacteroidetes and a decrease of Firmicutes. Treatment of lupus-prone mice with butyrate resulted in increased abundance of Firmicutes (*P* = 0.003), Clostridia (*P* = 0.005), Clostridiales (*P* = 0.005), *Lachnospiraceae* (*P* = 0.009), *Ruminococcaceae* (*P* = 0.021), *Peptostreptococcaceae* (*P* = 0.021), *Ruminiclostridium* (*P* = 0.016), *Oscillibacter* (*P* = 0.048), *Romboutsia* (*P* = 0.025), *Lachnoclostridium* (*P* = 0.012), *Coprococcus* (*P* = 0.015), *Ruminococcus* (*P* = 0.011), *Clostridium leptum* (*P* < 0.05), and *Dorea_spp*. (*P* = 0.019), and a reduced proportion of Bacteroidetes (*P* = 0.004), Bacteroidia (*P* = 0.004), and Bacteroidales (*P* = 0.004). Further, butyrate supplementation could ameliorate kidney damage. Overall, this study suggests that gut microbiota alterations occur in MRL/lpr lupus-prone mice following treatment with butyrate. Butyrate supplementation ameliorated gut microbiota dysbiosis. These findings support the use of butyrate and butyrate-producing bacteria as potential treatments for SLE.

## Introduction

Systemic lupus erythematosus (SLE) is a prototypic autoimmune disorder that damages multiple organs, including the kidneys, joints, skin, lung, heart, and brain (1). Although the pathogenesis of lupus is unclear, genetic predisposition as well as hormonal and environmental factors are involved. There is no cure, and treatment relies mainly on immunosuppressants. Although the symptoms are effectively managed with immunosuppressants, the side effects of these therapeutic drugs are concerning. Patients undergoing prolonged immunosuppression are more likely to experience higher infection rates and more serious infections. There is therefore an urgent need to explore the pathophysiological mechanisms of SLE and develop new treatment strategies.

Recent studies demonstrate that dysbiosis of the gut microbiome promotes autoimmune diseases such as inflammatory bowel disease, type 1 diabetes, rheumatoid arthritis, and multiple sclerosis (2). Growing evidence suggests that gut microbiota also plays a role in SLE (3). One cross-sectional study found dysregulated fecal microbiota with a reduced Firmicutes/Bacteroidetes ratio in patients with SLE (4). In an MRL/lpr lupus-prone mice model, the intestinal microbiota underwent a decrease in *Lactobacillaceae* and an increase in *Lachnospiraceae*. The indices of lupus disease severity (such as lymphadenopathy and glomerulonephritis) were found to be correlated with the abundance of *Lachnospiraceae* and *Lactobacillaceae* (5). *Lactobacillus* supplementation decreased levels of proteinuria and lupus autoantibodies, leading to amelioration of renal pathology of MRL/lpr mice (6). Further, *Lactobacillus fermentum* prevented vascular disorders and kidney damage in SLE mice (7, 8). However, it remains unclear whether these alterations in intestinal microbiome are causative or merely a result of disease status in SLE.

Short-chain fatty acids (SCFA), particularly acetate, propionate, and butyrate, are end products resulting from the intestinal bacterial fermentation of non-starch polysaccharides. The production of SCFAs relies on the commensal action of intestinal microbiota. Among SCFAs, butyrate is a main energy source for the colonic mucosa and increases the expression of tight junction proteins (TJPs) to better protect the gut barrier. At the intestinal level, butyrate ameliorates mucosal inflammation and oxidative status. At the extra-intestinal level, butyrate also benefits immune regulation and moderates genetic metabolic diseases, hemoglobinopathies, insulin resistance, hypercholesterolemia, and ischemic stroke (9).

Several studies have shown that butyrate can regulate T-independent and T-dependent antibody responses, as well as autoantibody responses, in lupus mouse models by directly influencing B-cell-intrinsic epigenetic mechanisms via the inhibition of histone deacetylases (HDACs) (10–12). In addition, intervention with a mixture of SCFAs consisting of sodium acetate, propionate, and butyrate decreased proteinuria and inhibited spleen enlargement in lupus-prone TLR7.1 Tg C57B1/6 mice (13). However, the effects of butyrate on gut microbiota in SLE have not yet been explored. In this study, we tested the effects of butyrate supplementation on gut microbiota dysbiosis and kidney damage in an MRL/lpr lupus-prone mice model.

## Materials and Methods

### Mice and Housing

Twelve 4–6 week-old MRL/lpr mice were obtained from Shanghai Laboratory Animal Co. Ltd. (SLAC, Shanghai, China). Eight sex- and age-matched BALB/C wild-type mice were provided by Guangdong Medical Lab Animal Center (GMLAC, Foshan, China). Female mice were used in this experiment, because lupus is more prevalent in females (1, 14). Mice were maintained in standard individually ventilated cages (IVCs) at the facility of GMLAC in specific pathogen-free (SPF) conditions. They were housed in constant temperature at 25 ± 1°C with 50 ± 5% humidity under a 12 h light/12 h dark cycle, and provided *ad libitum* with food and water. All animal experiments were conducted as required by the guidelines of the Institutional Animal Care and Use Committee of China.

### Butyrate Treatment and Sample Collection

The occurrence of autoimmune responses occurs as early as 6 weeks of age in lupus-prone MRL/lpr female mice (15, 16).To ascertain the effect of butyrate on active disease in MRL/lpr mice, mice were treated with butyrate starting at 8 weeks of age and after disease occurrence to mimic patients with active early-stage lupus. At the age of 8 weeks, female MRL/lpr mice were randomly separated into two groups (*n* = 6 per group): phosphate-buffered saline (PBS) -treated control group (MRL/lpr) and butyrate-treated group (MRL/lpr + Butyrate). Mice were weighted weekly and treated with sodium butyrate (Sigma-Aldrich, USA; 320 mg/kg, 0.1 mL/10 g body weight) or phosphate-buffered saline (PBS, 0.1 mL/10 g body weight). Treatments were administered three times per week (namely every Monday, Wednesday, and Friday) for 8 weeks by gavage, starting at 8 weeks of age until 16 weeks of age, when subjects were humanely euthanized. After the mice were killed, stool, blood, and kidney tissues were collected for further analysis.

### Detection of Fecal Butyrate Using Gas Chromatography-Mass Spectrometry (GC-MS)

Fecal samples were collected from 16 week-old BALB/C wild-type mice and MRL/lpr mice for butyric acid detection by GC-MS analysis. Fecal samples (200 mg) were sufficiently mixed with 2 mL of ultrapure water before centrifuging for 10 min at 13,000 rpm at 4°C. Then, 10 μL of 50% of sulfuric acid and 0.5 g of sodium sulfate were added to the supernatant followed by centrifugation at 6,000 rpm for 10 min. The ether layer was removed, and 50 μL of internal standard was added for GC-MS analyses according to a previously described method (17).

### Detection of Anti-dsDNA Ab and ANA

Serum was isolated from blood after clotting and stored at −20°C until use. Mouse anti-double stranded DNA antibodies (anti-dsDNA-Ab) and anti-nuclear antibodies (ANA) were detected using enzyme linked immunosorbent assay (ELISA) kits (mlbio, Shanghai, China) according to the manufacturers' instructions.

### Renal Histology

Kidneys were fixed in 4% neutral buffered formaldehyde and embedded in paraffin wax. Sections were stained with hematoxylin-eosin (H-E), periodic acid-Schiff (PAS), and Masson's Trichrome, and then examined by light microscopy (Olympus, Japan). Histological sections of the kidneys from each mouse were qualitatively and quantitatively assessed. Semi-quantitative evaluation of renal tissue was accomplished by scoring the damage severity according to previously published criteria (18, 19).

### Stool Sampling and Gut Microbiota Analysis

Stool samples were freshly collected by taking individual mice out of their cages. Samples were kept at −80°C. All fecal samples were processed at the same time. Samples were homogenized, lysed, and DNA was extracted as previously described (20, 21). PCR and 16S rRNA sequencing were conducted using MiSeq System (Illumina, Inc.). Bioinformatic analysis was performed as previously reported (20, 21). For statistical analysis, we used the unpaired Student's *t*-test and Wilcoxon test for comparison between two groups. One-way ANOVA and Tukey's post-test were performed to compare three groups. *P* < 0.05 was considered to indicate statistically significant results (^*^P < 0.05, ^**^P < 0.01, ^***^P < 0.005). All analyses were performed with Prism GraphPad 6.0.

## Results

### Butyrate Treatment Exerted Protective Effect in Terms of Disease Attenuation in MRL/lpr Mice

**Figure 1:**
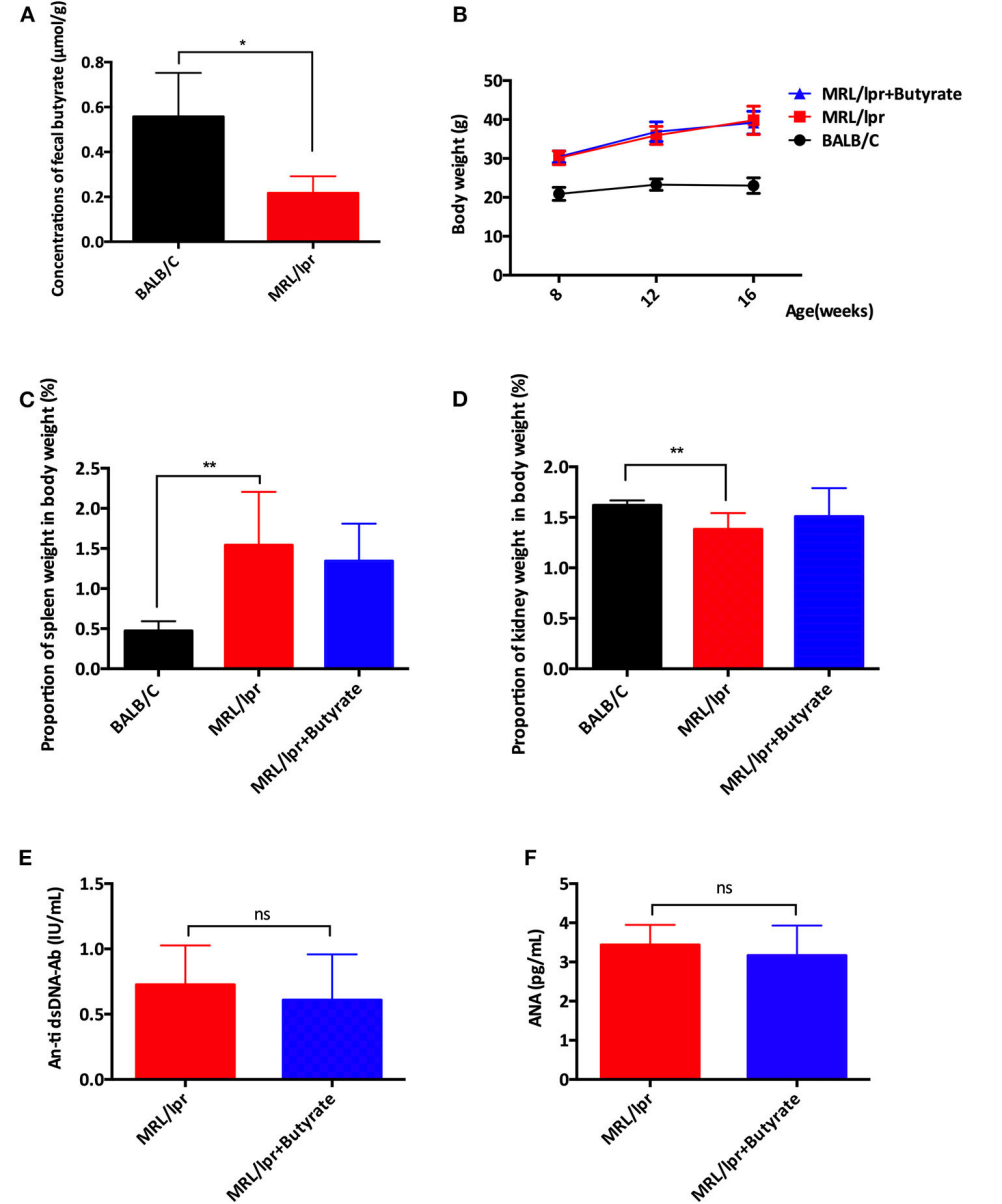
Butyrate treatment exerted protective effects by causing disease attenuation in female MRL/lpr mice. **(A)** The concentrations of butyrate in stool samples of BALB/c mice and MRL/lpr mice. **(B)** Body weights at weeks 8, 12, and 16. **(C)** Spleen weight to body weight ratio (%) at 16 weeks of age **(D)** Kidneys weight to body weight ratio (%) at 16 weeks of age in BALB/c mice, MRL/lpr mice, and MRL/lpr mice with butyrate treatment. **(E)** Serum ds-DNA-Ab levels and **(F)** Serum ANA levels of MRL/lpr mice at 16 weeks of age; *P < 0.05, **P < 0.01; ns, no significant difference.

**Figure 2:**
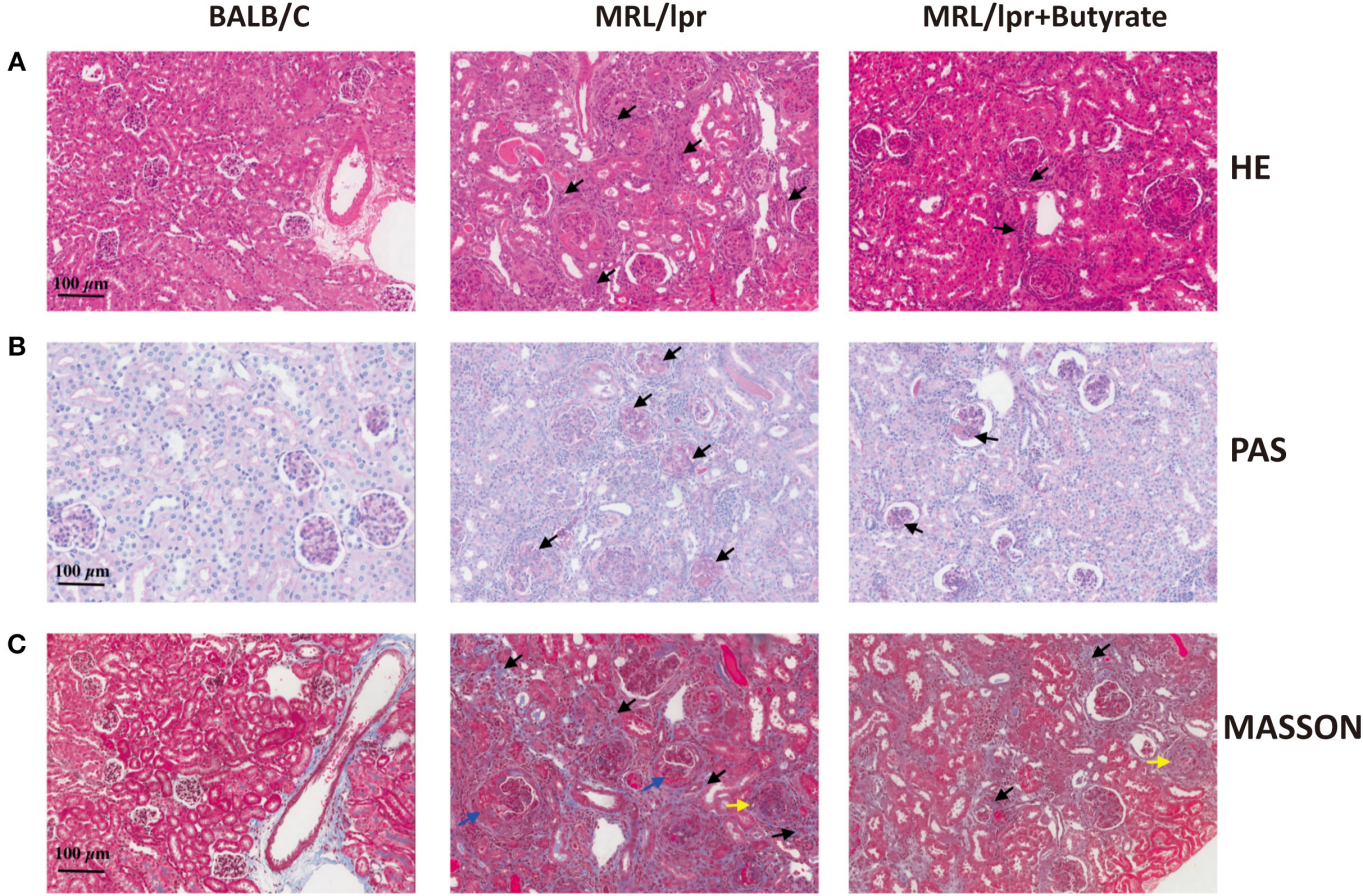
Effects of butyrate treatment improved renal histopathology of MRL/lpr mice at 16 weeks of age. HE staining **(A)**, PAS staining **(B)**, and Masson staining **(C)**. magnification, ×200; Bar equals 100 μm. Kidney pathological staining showed both proliferation of glomerular mesangial cells and mesangial matrix (arrow in PAS staining) as well as presence of glomeruli sclerosis (yellow arrow in the Masson staining figure) and crescents (blue arrow in the Masson staining figure) were observed in MRL/lpr lupus-prone mice. Lower levels of mesangial cell proliferation, mesangial matrix widening, and inflammatory cell infiltration (arrow in HE staining figure) and interstitial fibrosis (black arrow in Masson staining figure) were observed in the kidneys of mice treated with butyrate.

The effect of butyrate treatment on lupus was determined using the female MRL/lpr lupus-prone mouse model. MRL/lpr mice were gavaged with sodium butyrate or PBS starting from 8 weeks until 16 weeks of age. Compared with BALB/c mice (*n* = 8), MRL/lpr mice had lower fecal butyrate (mean ± SEM: 0.5567 ± 0.9778 vs. 0.2164 ± 0.04337 μmol/g, *P* < 0.05) and greater body weight gains (Figures 1A,B). One mouse in the MRL/lpr model group was found dead at week 11, while no deaths occurred in the BALB/c group and butyrate-treated MRL/lpr group (data not shown). Spleen weight to body weight ratio (%) was higher in MRL/lpr lupus-prone mice than in BALB/c mice, indicating immune system hyperactivity in SLE Figure 1C. The ratio of the kidney weight to body weight was lower in the MRL/lpr lupus-prone mouse model group than in BALB/c mice, suggesting the presence of kidney atrophy and glomeruli sclerosis (Figure 1D). Interestingly, renal histopathological changes were alleviated in the butyrate-treated MRL/lpr mice compared with the MRL/lpr lupus-prone model mice as shown in Figure 2. Specifically, kidney pathological staining showed an absence of renal lesions in the BALB/c mice, while both proliferation of glomerular mesangial cells and mesangial matrix (arrow in the Figure 2B showing PAS staining) and presence of glomeruli sclerosis (yellow arrow in the Figure 2C showing Masson staining) and crescents (blue arrow in the Figure 2C showing Masson staining) were observed in MRL/lpr lupus-prone mice. Compared with the MRL/lpr lupus-prone model group, a lower degree of mesangial cell proliferation with less mesangial matrix widening and reduced inflammatory cell infiltration (arrow in the Figure 2A showing HE staining) and interstitial fibrosis (black arrow in the Figure 2C showing Masson staining) were observed in kidneys from mice treated with butyrate Figure 2. However, we did not observe obvious differences after butyrate treatment in anti-dsDNA-Ab (mean ± SEM: 0.7260 ± 0.1346 vs. 0.6080 ± 0.1568 IU/mL, *P* = 0.7749) and ANA level (mean±SEM:3.437 ± 0.2095 vs. 3.164 ± 0.3436 pg/mL, *P* = 0.3995) relative to mice treated with PBS in this study Figures 1E,F.

### Butyrate Treatment Increased Microbial Diversity in MRL/lpr Mice

**Figure 3:**
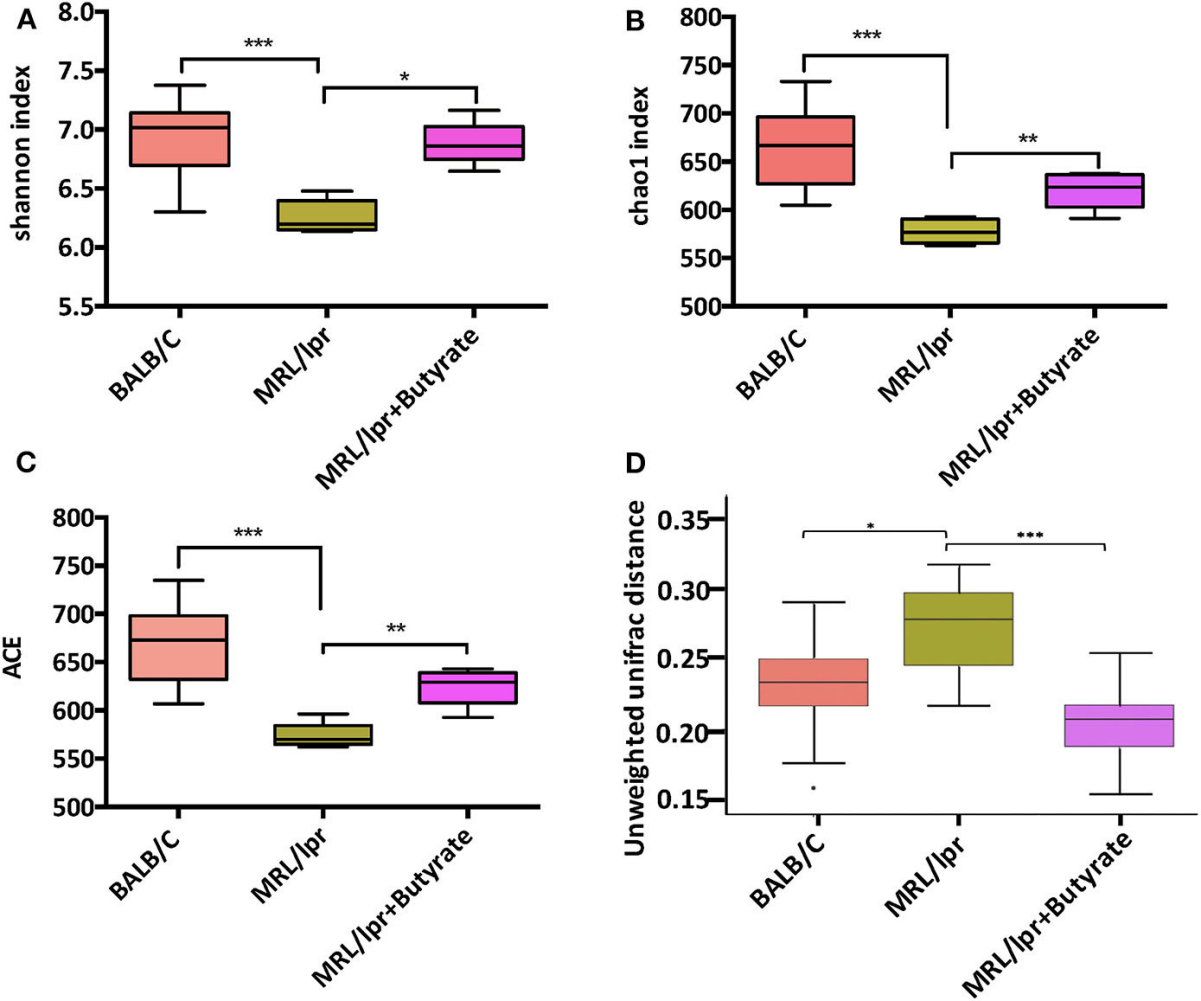
Butyrate treatment elicited an increase in gut microbial diversity. Shannon index **(A)**, Chao1 index **(B)**; ACE index **(C)**, and unweighted Unifrac distance **(D)**. Wilcoxon test was utilized to compare two groups; *P < 0.05, **P < 0.01, ***P < 0.005.

To investigate the general differences in gut microbiota, α- and β diversity were evaluated to assess bacterial richness and evenness. A marked decrease in microbial diversity as indicated by the Shannon index (*P* < 0.005), chao1 (*P* < 0.005), and ACE index (*P* < 0.005), was found in MRL/lpr mice than those in BALB/c mice Figures 3A–C. Compared to PBS-treated MRL/lpr mice, butyrate-treated MRL/lpr mice elicited a marked increase in microbial α-diversity Figures 3A–C. In regard to β-diversity, unweighted UniFrac distance results showed a clear separation of the community composition between the three groups Figure 3D.

### Butyrate Treatment Improved Gut Microbiota Composition in MRL/lpr Mice

**Figure 4:**
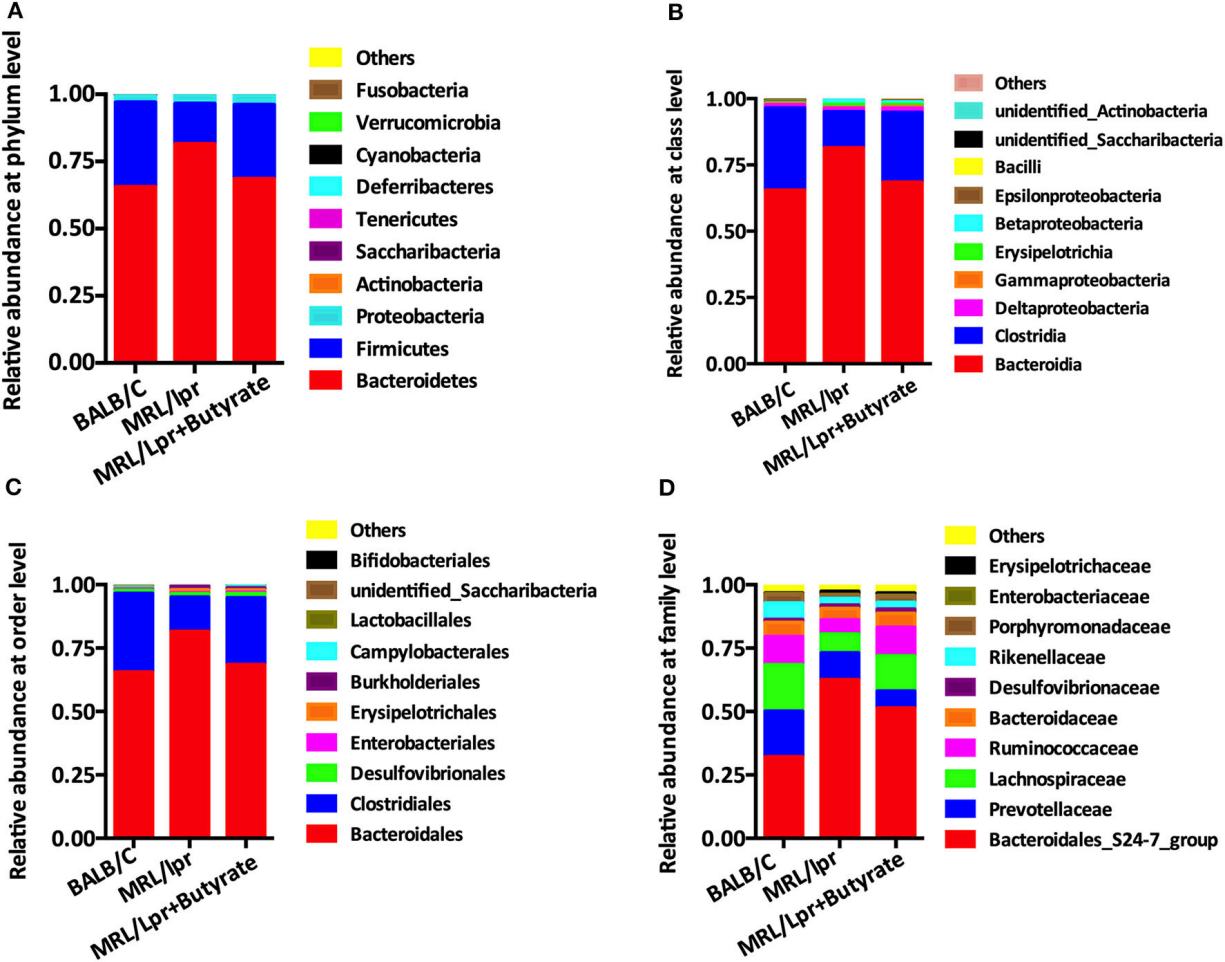
Butyrate treatment altered the composition of intestinal microbiome. **(A)** Relative abundance at the phylum level. **(B)** Relative abundance at the class level. **(C)** Relative abundance at the order level. **(D)** Relative abundance at the family level.

Based on the observed differences in α- and β-diversity, we further explored the differences in the relative abundance of bacterial taxa. Compared with that in BALB/c control mice, the intestinal microbiota of MRL/lpr mice had a significantly lower level of Firmicutes at the phylum level Figure 4A, Clostridia at the class level Figure 4B, Clostridiales at the order level Figure 4C, and *Lachnospiraceae* at the family level Figure 4D, with a higher abundance of Bacteroidetes, Bacteroidia, and Bacteroidales Figures 4A–C. However, it was unclear whether this alteration of gut microbiota was a cause or a result of disease onset. After 8 weeks of butyrate treatment in MRL/lpr mice, the relative abundance of Firmicutes, Clostridia, Clostridiales, and Lachnospiraceae was significantly increased, while the levels of Bacteroidetes, Bacteroidia, and Bacteroidales were significantly reduced than those in PBS-treated MRL/lpr mice Figures 4A–D.

**Figure 5:**
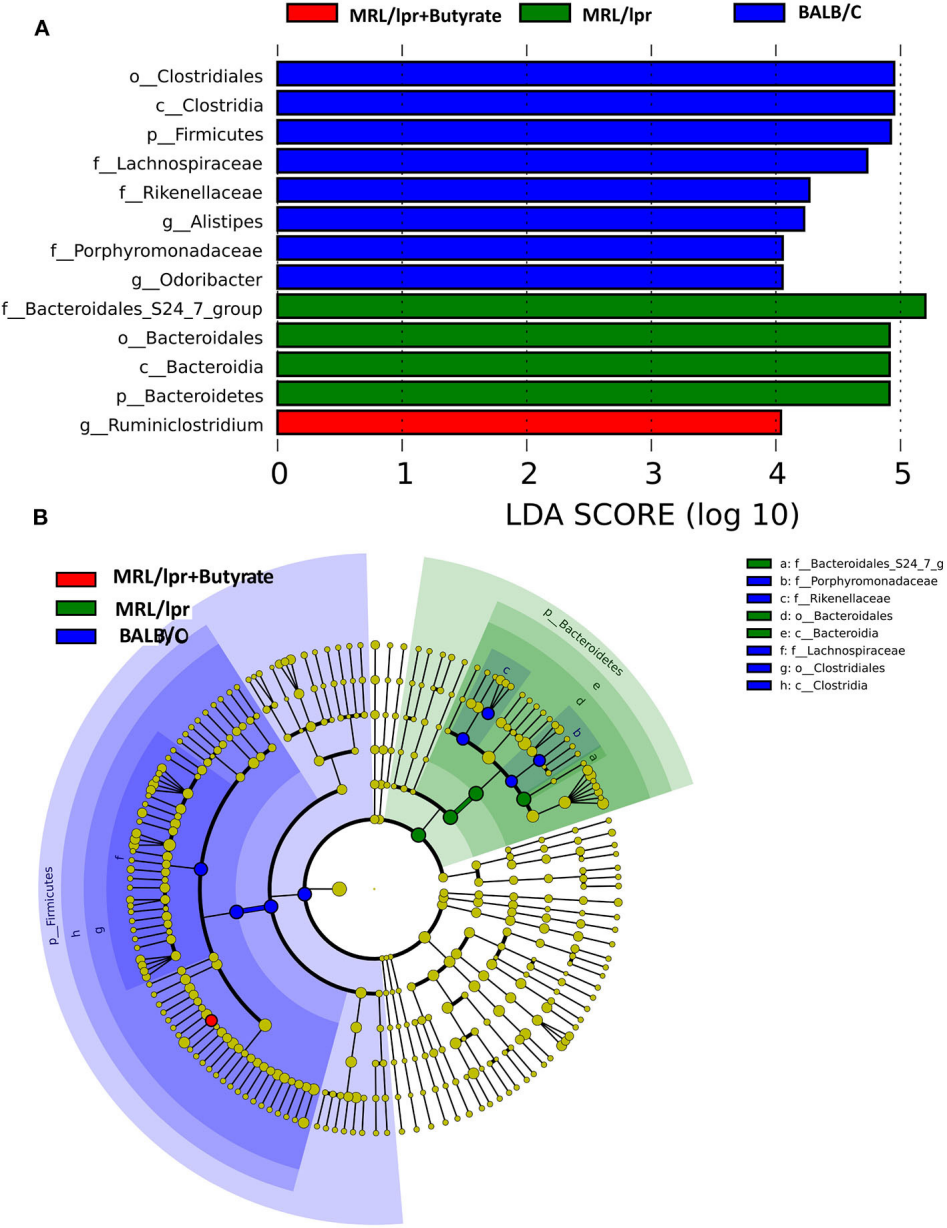
Taxonomic biomarker identification in gut microbiota by linear discriminant analysis (LDA) effect size (LEfSe) and LDA analysis. LEfSe was used to identify differentially abundant taxa **(A)**, and the LDA score **(B)**. Significantly distinct taxa among the BALB/c mice (blue), MRL/lpr mice (green), and butyrate-treated MRL/lpr mice (red).

**Figure 6:**
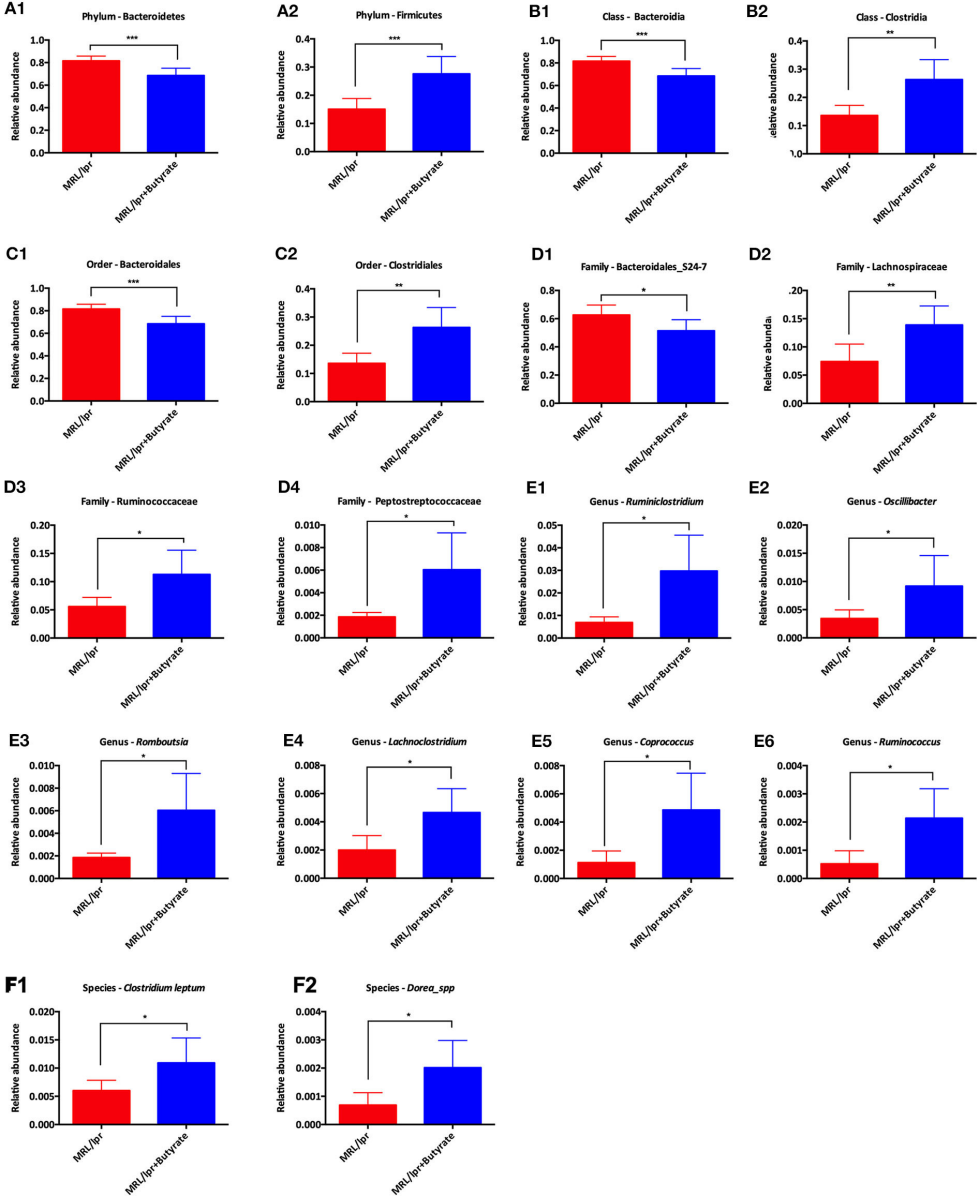
*T*-test bar plot for analysis of difference in taxa from phylum level to species level between MRL/lpr mice and butyrate-treated MRL/lpr mice. Analysis of taxa differences at the phylum level **(A)**, at the class level **(B)**, at the order level **(C)**, at the family level **(D)**, at the genus level **(E)**, and at the species level **(F)**; *P < 0.05, **P < 0.01, ***P < 0.005.

Linear discriminant analysis (LDA) effect size (LEfse) showed that genera belonging to phylum Bacteroidetes were significantly increased in PBS-treated MRL/lpr control mice, while genus *Ruminiclostridium* at phylum Firmicutes was markedly enriched in mice treated with butyrate (LDA > 4, *P* < 0.05) Figure 5. Next, we investigated significant taxonomic shifts in the microbial community using *T*-test bar plot analysis from the phylum to species level. Interestingly, the proportions of Firmicutes (*P* = 0.003), Clostridia (*P* = 0.005), Clostridiales (*P* = 0.005), *Lachnospiraceae* (*P* = 0.009), *Ruminococcaceae* (*P* = 0.021), *Peptostreptococcaceae* (*P* = 0.021), *Ruminiclostridium* (*P* = 0.016), *Oscillibacter* (*P* = 0.048), *Romboutsia* (*P* = 0.025), *Lachnoclostridium* (*P* = 0.012), *Coprococcus* (*P* = 0.015), *Ruminococcus* (*P* = 0.011), *Clostridium leptum* (*P* < 0.05), and *Dorea_spp*. (*P* = 0.019) were significantly increased, and Bacteroidetes (*P* = 0.004), Bacteroidia (*P* = 0.004), and Bacteroidales (*P* = 0.004) were markedly decreased after butyrate treatment Figure 6. Overall, these data strongly suggest that microbiota-derived butyrate alters the composition of the fecal microbiome.

## Discussion

Alteration of gut microbiota influences the prognosis of systemic lupus erythematosus (SLE). Butyrate, which is generated by the fermentation of dietary fiber by intestinal microbiota, is recognized for its therapeutic potential in chronic diseases, including inflammatory bowel disease (IBD), cancer, inherited diseases, and neurological degenerative disorders (22). Butyrate, which functions as a histone deacetylase inhibitor, has also been shown to upregulate B cell microRNAs and modulate antibody and autoantibody responses through its direct effects on B-cell-intrinsic epigenetic mechanisms in lupus mouse (10–12). In addition, butyrate-generating bacteria *Lactobacillus fermentum* exerted beneficial effects in terms of reducing lupus disease activity and renal damage (7, 8). However, the effects of butyrate on gut microbiota in SLE are not yet understood. In present study, we investigated the protective effects of butyrate on intestinal microbiome in the lupus-prone MRL/lpr mouse model.

The intestinal microbial composition of lupus-prone MRL/lpr mice was consistent with the shifts observed in SLE patients; in particular, an increased abundance of members of Bacteroidetes and a decrease in those of Firmicutes were found (4). In this study, butyrate treatment significantly increased the microbial diversity and abundance of Firmicutes and markedly reduced that of Bacteroidetes, Bacteroidia, and Bacteroidales. An increased proportion of members of phylum Firmicutes has been found to be associated with beneficial metabolic profile and energy intake (23). Butyrate improves energy metabolism by reducing energy intake and increasing fat oxidation through the activation of brown adipose tissue (24), and caloric restriction has been shown to prevent the progression of lupus-like disease in NZB and (NZBxNZW) F1 mice (25, 26). Moreover, although the production of butyrate is dependent on diet and intestinal microflora composition, butyrate also modulates intestinal microflora through regulation of gut lumen pH and exerts many beneficial extra-intestinal effects through epigenetic mechanisms (9). Mildly acidic pH values are beneficial for butyrate-producing bacteria by enabling them to compete against gram-negative carbohydrate-utilizing bacteria such as *Ba*c*teroides* spp. (27). Moreover, butyrate suppresses inducible nitric oxide synthase (iNOS) synthesis in the gut by activating PPAR-γ-signaling in colonocytes, thus preserving epithelial hypoxia and limiting the growth of nitrate respiration-dependent dysbiotic to maintain gut homeostasis (28, 29). A recent study (30) demonstrated the effectiveness of helminth-derived tuftsin and phosphorylcholine for treating mice with lupus and demonstrated its association with an alteration in gut microbiota. In their study, butyrate-producing bacteria were associated with the amelioration of disease following treatment. Taken together, these findings illustrate the possible mechanisms by which effective SLE treatment influences the microbiome by promoting the growth of beneficial microbial communities.

Anti-dsDNA antibodies facilitate the inflammatory processes involved in tubulointerstitial nephritis by binding to the renal proximal tubular epithelial cells and inducing pro-inflammatory mediators (31). In SLE, excessive albumin is filtered through glomeruli and reabsorbed in proximal tubular cells. This leads to a deleterious inflammatory cycle as a result of the secretion of pro-inflammatory cytokines, and subsequent infiltration of inflammatory cells (32). Recently, Sanchez et al. (10) confirmed that dietary fiber-derived SCFAs reduced the autoantibody response (ANA, anti-dsDNA, anti-histone, anti-RNP/Sm, and anti-RNA IgG1 and IgG2a) and autoimmunity in lupus-prone mice. In our study, we found no significant difference in anti-dsDNA ab and ANA levels after butyrate treatment. Interestingly, butyrate supplementation alleviated kidney damage in this study. Various immune cells, especially dysfunctional T and B cells, have been shown to have harmful effects in SLE (33, 34). The imbalance between inflammatory T-helper (Th17) cells and anti-inflammatory regulatory T (Treg) cells has been implicated in the pathogenesis of SLE (35). Butyrate can decrease the differentiation of Th17 cells by inhibiting histone deacetylase 3 (36), which may in turn attenuate lupus. In addition, butyrate has also been shown to contribute to CD4 + Foxp3 + T cell induction *in vitro* (37), suggesting that this compound may attenuate lupus by altering the balance between Th17 cells and Treg cells. Furthermore, “leaky” gut, which is associated with gut dysbiosis, has been recognized to occur in the onset of both murine lupus and human SLE (6, 38). Butyrate has been shown to restore the mucosal barrier function in many disorders related to leaky gut (39–41). Therefore, butyrate may alleviate kidney pathology and ameliorate lupus by enhancing the intestinal epithelial barrier and protecting against the translocation of LPS and/or LPS-producing bacteria from the intestinal lumen to the circulation, even to the kidney. Given the chronic nature of SLE, it is not easy to reduce autoantibody levels through short-term treatments or a single intervention. Improved knowledge of these compounds and combined treatments may enhance our understanding of disease etiology and optimize treatment protocols. Overall, our novel data also showed that butyrate treatment significantly ameliorated gut kidney damage. These findings support the role of butyrate and butyrate-producing bacteria in the onset and development of SLE.

However, some limitations of this study should be noted. We did not compare the dynamic and longitudinal changes of gut microbiota across the 8, 12, and 16 week time points. Additionally, more consideration should be given to gut-derived direct signaling mechanisms. More optimized study design, a larger sample size, and different sampling times would benefit future studies. The current study demonstrates the potential effects of butyrate on gut microbiota and kidney damage in mice. The immunomodulatory activity of butyrate was associated with alterations in the composition of the intestinal microbiome, including increases in beneficial bacteria and decreases in bacteria that promote inflammation. The activity of intestinal microbiota may act as another potential mechanism contributing to the immunomodulatory effect of butyrate in mice with lupus. Altogether, the present study reveals that butyrate treatment induces several alterations in the gut microbiota of lupus-prone mice. In addition, butyrate supplementation was shown to ameliorate kidney damage in this study. Further studies are required to address the mechanisms underlying the effects of butyrate against lupus, which would provide further evidence of the immunomodulatory activity of butyrate as a potential therapeutic strategy for SLE.

## Data Availability Statement

The datasets presented in this study can be found in NCBI-SRA under accession number PRJNA663085.

## Ethics Statement

The animal study was reviewed and approved by Guangdong Medical Lab Animal Center.

## Author Contributions

HH and HX conducted the study, collected the data, and wrote the manuscript. JX, HZ, and QL interpreted the results and prepared the figures. YZ and YN directed the project, interpreted the results, and approved the final manuscript. All authors contributed to the article and approved the submitted version.

## Conflict of Interest

The authors declare that the research was conducted in the absence of any commercial or financial relationships that could be construed as a potential conflict of interest.
